# Candidiasis oral mixta en pacientes con diabetes de tipo 2: identificación y espectro de sensibilidad

**DOI:** 10.7705/biomedica.6878

**Published:** 2023-08-31

**Authors:** Javier Araiza, Valentín Sánchez-Pedraza, Ana Karen Carrillo, Denise Fernández-Samar, Jazmín Tejeda, Alexandro Bonifaz

**Affiliations:** 1 Laboratorio de Micología, Servicio de Dermatología, Hospital General de México “Dr. Eduardo Liceaga”, Ciudad de México, México Laboratorio de Micología Servicio de Dermatología Hospital General de México “Dr. Eduardo Liceaga” Ciudad de México México; 2 Servicio de Endocrinología, Hospital General de México “Dr. Eduardo Liceaga”, Ciudad de México, México Servicio de Endocrinología Hospital General de México “Dr. Eduardo Liceaga” Ciudad de México México

**Keywords:** diabetes mellitus de tipo 2, candidiasis, Candida, Candida albicans, Diabetes mellitus, type 2, candidiasis, Candida, Candida albicans

## Abstract

**Introducción.:**

Los pacientes con diabetes mellitus de tipo 2 son propensos a adquirir infecciones por *Candida* spp., en ocasiones, causadas por más de una especie. La resistencia de algunas de ellas puede resultar en complicaciones médicas por falla del tratamiento.

**Objetivos.:**

Determinar la frecuencia y las variedades clínicas de la candidiasis oral mixta en pacientes con diabetes mellitus de tipo 2, las especies de *Candida* involucradas y sus espectros de sensibilidad a los antifúngicos utilizados como tratamiento.

**Material y métodos.:**

Se hizo un estudio transversal analítico en pacientes con diabetes mellitus de tipo 2, hiperglucemia (superior o igual al 7 % de la hemoglobina glucosilada, HbA1C) y con diagnóstico clínico de candidiasis oral. Mediante técnicas microbiológicas, se identificaron las especies causales de la candidiasis oral. Las pruebas de sensibilidad se llevaron a cabo con el método de difusión en placa con tiras (E-test^®^).

**Resultados.:**

Se incluyeron 72 pacientes: 32 (44 %) hombres y 40 (56 %) mujeres, clasificados en tres grupos de edad: jóvenes adultos (17 %), adultos (74 %) y ancianos (9 %), con una media de 51 años. No se encontraron diferencias significativas en la candidiasis oral según los grupos de sexo y edad, ni entre las candidiasis orales mixtas y el sexo, el porcentaje de HbA1C, el tratamiento antihiperglucemiante o el tiempo de diagnóstico de la diabetes mellitus de tipo 2. En el grupo etario de adultos, se encontró una correlación con las candidiasis mixtas o simples. Se encontraron 8 (13 %) casos de candidiasis mixtas: siete con coinfección por dos especies de *Candida* y uno con coinfección por tres especies. Las especies identificadas en ellos, fueron: *Candida albicans*, *C. glabrata*, *C. dubliniensis*, *C. kefyr*, *C. tropicalis* y *C. krusei*. La mayoría de estas especies presentó sensibilidad a ketoconazol y fluconazol, y mayor resistencia a itraconazol.

**Conclusiones.:**

Las candidiasis orales mixtas se presentan, aproximadamente, en el 10 % de los pacientes con diabetes mellitus de tipo 2 y el tratamiento puede ser ineficaz cuando no se identifica el agente etiológico.

## Abstract (en)

**Introduction.:**

Patients with type 2 diabetes mellitus are susceptible to acquire *Candida* spp. infections, sometimes involving more than one species. The resistance of some species to antimycotic agents can cause treatment failure.

**Objectives.:**

To determine the frequency and clinical varieties of mixed oral candidiasis in patients with type 2 diabetes mellitus, the involved species, and its sensitivity spectra when exposed to antifungals used as candidiasis treatment.

**Material and methods.:**

We developed an analytical cross-sectional study with 72 patients with type 2 diabetes mellitus with hyperglycemia (HbAIC s 7%) and an oral candidiasis diagnosis. The causal species of oral candidiasis were identified through microbiological techniques, and sensitivity tests were carried out using the diffusion method in a plate with strips (E-test ®).

**Results.:**

We included 72 patients in the study, 32 (44%) males and 40 (56%) females. Patients were divided into three age groups: young adults (17%), adults (74%), and older adults (9%). The mean age of the patients was 51 years. No significant differences were found between mixed oral candidiasis and groups (sex and age), or between mixed oral candidiasis and gender, glycosylated hemoglobin level (HbA1C), antihyperglycemic treatment, or type 2 diabetes mellitus time of diagnosis. We found a correlation between the adult group and development of mixed or simple oral candidiasis. The results showed eight (13%) cases of mixed oral candidiasis: seven with a coinfection of two species and one with a coinfection of three species. The identified species were *Candida albicans*, *C. glabrata*, *C. dubliniensis*, *C. kefyr*, *C. tropicalis*, and *C. krusei*. Most of these species presented sensitivity against ketoconazole and fluconazole, and higher resistance to itraconazole.

**Conclusions.:**

Mixed oral candidiasis occurs in approximately 10% of the patients with type 2 diabetes mellitus and its treatment can be ineffective when the etiological agent is not identified.

La diabetes mellitus de tipo 2 es una enfermedad multisistémica, de disfunción metabólica, que se caracteriza por un aumento en la concentración de glucosa en la sangre debido a un déficit absoluto o relativo de insulina, el cual induce alteraciones en el metabolismo de los carbohidratos, los lípidos y las proteínas. Sin embargo, esta enfermedad resulta de la interacción entre una predisposición genética, y factores de riesgo conductuales y ambientales ^1^^,^^2^.

En México, el Instituto Nacional de Estadística y Geografía (INEGI) reportó que, en el 2018, el porcentaje de habitantes con diabetes mellitus era de 10,3 % ^3^, lo que convierte a la diabetes mellitus de tipo 2 en uno de los problemas de salud pública más importantes de la actualidad. En las últimas décadas, han aumentado constantemente el número de casos y la prevalencia de la enfermedad ^4^.

Las complicaciones agudas -que aparecen por un mal control de la enfermedad- y las crónicas -que surgen conforme va evolucionando- son factores de morbimortalidad en esta población ^5^.

Con mayor frecuencia, los pacientes presentan estados hiperosmolares y complicaciones microvasculares y macrovasculares irreversibles, que incluyen: retinopatía, nefropatía y neuropatía; además de enfermedades cardiovasculares como infarto agudo al miocardio, enfermedad vascular cerebral e insuficiencia arterial periférica ^6^.

En el paciente diabético, también se presentan complicaciones en la cavidad bucal que, aunque no son específicas o patognomónicas, sí son más frecuentes y de peor evolución; entre estas se destacan: caries dental, glositis romboidal media, xerostomía, síndrome de ardor bucal, agrandamiento de las glándulas salivales, disgeusia, procesos infecciosos, etc. ^7^.

En estos últimos, la incidencia de infecciones fúngicas en pacientes con diabetes mellitus de tipo 2 se reconoció hace muchos años ^8^. La candidiasis oral es la infección más frecuente y es producida por hongos levaduriformes oportunistas, del género *Candida*^9^. Estas levaduras son responsables de la mayor parte de las infecciones fúngicas en pacientes inmunosuprimidos. Las especies patógenas más comunes son: *C. albicans*, *C. glabrata*, *C. parapsilosis*, *C. tropicalis* y *C. krusei*^10^^,^^11^. En un estudio realizado en Latinoamérica por Suárez *et al*., en 2013, sobre candidiasis oral en pacientes con diabetes mellitus de tipo 2, se encontró que la especie predominante era *C. albicans* (30 %). Estos autores reportaron una frecuencia del 4,9 % de candidiasis mixtas causadas principalmente por *C. krusei* y *C. albicans*, *C. glabrata* y *C. albicans*^12^.

Por otra parte, Mohammadi *et al*. ^13^ reportaron que de 58 pacientes con diabetes de tipo 2, el 55 % fue positivo para candidiasis oral, causada por especies del género *Candida*, como *C. albicans* (36,2 %), *C. krusei* (5,2 %), *C. glabrata* (3,4 %) y *C. tropicalis* (3,4 %). Es importante señalar que también se identificaron varias de estas especies en una misma muestra, lo cual indica una candidiasis mixta. 

La Organización Mundial de la Salud reporta que las lesiones bucales localizadas suelen responder a los preparados tópicos, como el gel de miconazol o las suspensiones bucales de nistatina o anfotericina B. Sin embargo, solo se hace referencia a la posología para pacientes con HIV/sida pero no para las lesiones en aquellos con enfermedades en aumento, como la diabetes mellitus ^14^.

Existen varios agentes antifúngicos (azoles y polienos) con diferentes modos de acción, disponibles para el tratamiento de la infección oral por *Candida* spp.

La disponibilidad creciente y la aplicación clínica de estos agentes antifúngicos, en especial los tópicos, permite su uso para la prevención de infecciones por *Candida* spp. en pacientes con un control metabólico deficiente, particularmente en las personas mayores que usan dentaduras postizas. Estas personas a menudo sufren de una sensación desagradable en la boca que puede provocar angustia intolerable y si, además, son pacientes con diabetes mellitus de tipo 2 y un control glucémico deficiente, los médicos les prescriben agentes antimicóticos tópicos para prevenir la candidiasis oral ^15^. Dicho tratamiento, para personas diabéticas y no diabéticas, se basa en el uso de agentes antifúngicos tópicos, como azoles (principalmente miconazol y fluconazol, pero también, ketoconazol e itraconazol) o polienos (anfotericina B y nistatina) ^14^^,^^15^.

El uso indiscriminado de estos antimicóticos en México resulta en una mayor resistencia antifúngica, lo que complica la elección del tratamiento y su eficacia en las candidiasis mixtas ^16^.

El objetivo del estudio fue determinar la frecuencia de candidiasis oral mixta en pacientes con diabetes mellitus de tipo 2 e hiperglucemia (mayor o igual a 7 % de hemoglobina glucosilada). Está demostrado que alrededor del 5 % de las candidiasis, en cualquier forma clínica, son mixtas ^17^ y poseen diversos espectros de sensibilidad y factores de virulencia, que impactan en la respuesta terapéutica y las presentaciones clínicas.

## Materiales y métodos

Se llevó a cabo un estudio transversal analítico de pacientes hospitalizados en el área de Infectología del Hospital General de México “Dr. Eduardo Liceaga” con diabetes mellitus de tipo 2 e hiperglucemia (mayor o igual a 7 % de HbA1C). Se incluyeron pacientes adultos con diagnóstico de diabetes mellitus de tipo 2 y datos clínicos indicativos de candidiasis oral, que aceptaron participar en el estudio y firmaron el consentimiento informado. Se excluyeron aquellos con signos clínicos de candidiasis oral, pero con estudios micológicos sin estructuras visibles en el análisis microscópico, ni agentes identificados.

A los pacientes incluidos, se les examinó la cavidad oral y se les tomaron muestras mediante raspado de las lesiones orales. Se hizo un examen microscópico para buscar estructuras micóticas, pseudohifas, blastoconidios o ambos; y un cultivo en medio agar dextrosa de Sabouraud. Posteriormente, se hizo la resiembra en medios cromogénicos (CHROMagar^®^ *Candida*) para la búsqueda intencionada de más de una especie de *Candida*, mediante el desarrollo de colonias de diferentes colores. Las especies se identificaron mediante pruebas fisiológicas (características micromorfológicas en agar harina de maíz y 1 % de *Tween* 80) y bioquímicas (Auxacolor2 de BIO-RAD®).

Se practicaron pruebas de sensibilidad mediante la técnica de difusión en placa de agar de Müeller-Hinton, con suplemento de glucosa al 2 % y 0,<ml de azul de metileno, y en tiras de tipo E-test®, de acuerdo con las modificaciones al protocolo M44-A sobre el uso de disco antifúngicos de difusión del *Clinical and Laboratory Standards Institute*, realizadas por Pfaller *et al.*^18^. Se inocularon placas de agar de Müeller-Hinton de 15 cm de diámetro, con una suspensión de levaduras ajustada a 0,5 según la escala de McFarland (1 x 10^6^ - 5 x 10^6^ UFC/ml), mediante técnica masiva, con hisopo y rotando en tres direcciones, a fin de distribuir uniformemente el inóculo. Las placas se dejaron secar de 3 a 5 minutos, a fin de permitir la absorción de cualquier exceso de inóculo. En la superficie del agar inoculado, se colocaron tres tiras de E-test con distintos tipos de antimicóticos, cuidando de colocar el extremo de mayor concentración hacia el borde externo de la placa. Las placas se incubaron en aerobiosis a 35 °C durante 24 horas, para la posterior lectura de las ojivas de inhibición. El punto de intersección de la elipse de inhibición de crecimiento con la tira se tomó como la concentración mínima inhibitoria.

Se expusieron las 17 levaduras, identificadas en los casos de candidiasis oral mixta, a los antimicóticos sistémicos utilizados con más frecuencia para el tratamiento de dicha enfermedad: fluconazol, itraconazol y ketoconazol. El gradiente de concentración de las tiras fue de 4-64 μg/ml para el fluconazol, de 0,1-1,0 μg/ml para el itraconazol y de 0,12,0 μg/ml para el ketoconazol. Se utilizó como control de calidad la cepa de referencia *C. albicans* ATCC 10231 (no se contó en su momento con la cepa de *C. albicans* 90028), que ya tenía los puntos de corte de sensibilidad frente a los antimicóticos probados en el presente estudio. La sensibilidad de las cepas se reportó como: sensible, resistente o sensible según la dosis.

Los resultados obtenidos se recolectaron en una hoja de cálculo del programa Microsoft Excel, para su posterior manejo estadístico con ayuda del paquete computacional SPSS™, versión 24, bajo intervalos de confianza del 95 % y niveles de significación del 5 % (cuadro 1).

**Cuadro 1:** Inferencia de la relación entre la frecuencia de candidiasis oral mixta* con las variables analizadas

	Tipo de variable	Prueba estadística	^p^	Conclusión
Diferentes sectores etarios (jóvenes-adultos, adultos y ancianos	Cualitativa	Tabla de contingencia 2 x 2 - Ji al cuadrado de Pearson	0,698	Se acepta la hipótesis nula que establece la independencia entre ambas variables. No existe asociación.
Sexo (femenino, masculino)	Cualitativa	- Tabla de contingencia 2 x 2 - Ji al cuadrado y prueba de Fisher	0,461	Se acepta la hipótesis nula. No existe relación entre ambas variables.
Porcentaje de hemoglobina glucosilada (HbAIC)	Cuantitativa1 (p=0,2)			Se acepta la hipótesis nula. No existe relación entre ambas variables.
Tiempo de diagnóstico de diabetes mellitus de tipo 2 (años)	Cuantitativa2 (p=0,006)	t de Student	0,1	Se acepta la hipótesis nula. No existe relación entre ambas variables.
Tratamiento empleado como control de hiperglucemia	Cualitativa	Ji al cuadrado de Pearson	0,097	Se acepta la hipótesis nula. No existe relación entre ambas variables.

* Variable cualitativa

1 Distribución normal

2 Distribución no paramétrica

El trabajo fue sometido y aceptado por los comités de ética e investigación del Hospital General de México “Dr. Eduardo Liceaga”, con el número DI/18/109/04/097.

## Resultados

Se incluyeron 72 pacientes con diabetes mellitus de tipo 2 descontrolada, provenientes del Hospital General de México “Dr. Eduardo Liceaga”, 64 (89 %) estaban hospitalizados y 8 (11 %) asistieron a la consulta externa (no hospitalizados). De los 72 pacientes, 32 (44 %) eran hombres y 40 (56 %) mujeres, con rangos de edad entre 22 y 85 años (mediana de 54 años**).**

Para el análisis final, se incluyeron 63 pacientes: 62 con candidiasis pseudomembranosa, y uno de ellos, además, con queilitis angular, en los cuales se comprobó la infección oral mediante análisis microscópico de las muestras obtenidas de las lesiones de la cavidad bucal (cúmulos de blastoconidios, seudohifas o ambos). Los pacientes excluidos fueron los nueve en los que no se encontraron estructuras micóticas.

Los pacientes incluidos estaban conformados por 25 (39 %) hombres y 38 (61 %) mujeres, divididos en tres grupos distintos de edad, denominados como: jóvenes adultos, de 20 a 35 años (17 %), adultos, de 36 a 59 años (74 %), y ancianos, de 75 a 90 años (9 %).

Respecto a la relación entre candidiasis oral mixta y grupo etario, no se encontró ninguna asociación a que no existía diferencia estadísticamente significativa entre los grupos y el hallazgo de candidiasis oral (p=0,698). Lo mismo se estableció para el caso de candidiasis oral mixta y sexo (p=0,461).

Al analizar la posible relación entre los valores de hemoglobina glucosilada (HbA1C) y la presencia de candidiasis oral mixta, no se encontraron diferencias estadísticamente significativas entre el grupo con candidiasis oral simple (10,84 % de HbA1C) y aquel con candidiasis oral mixta (12,06 % de HbA1C) (p=0,1). Este comportamiento también se establece entre la detección de candidiasis oral mixta o candidiasis oral simple, y el tiempo de diagnóstico de la diabetes mellitus de tipo 2 (27,06 y 32,72 años, respectivamente) (p=0,412). Asimismo, se analizó si el tratamiento antihiperglucemiante influía sobre la presencia de candidiasis oral mixta o simple. Al evaluar la frecuencia de casos y el tratamiento, no se encontró una relación significativa (p=0,097); solo se observó que la mayoría de los casos se manejaban con insulina NPH: el 44,4 % (28/63) de las candidiasis orales por una especie y el 4,76 % (3/63) de las orales mixtas, y con metforminaglibenclamida, el 14,28 % (9/63) de las candidiasis orales por una especie y el 4,76 % (3/63) de las orales mixtas (cuadro 1).

Se identificaron ocho aislamientos mixtos que corresponden al 13 % de los pacientes incluidos; en siete de ellos se identificaron dos especies de *Candida* y en un paciente se encontraron tres especies; en seis casos estuvo implicada *C. albicans* (cuadro 2).

**Cuadro 2:** Especies de *Candida* identificadas en los pacientes con candidiasis oral mixta

Paciente	Especies identificadas
1	*Candida albicans* *Candida krusei* *Candida glabrata*
2	*Candida albicans* *Candida tropicalis*
3	*Candida albicans* *Candida kefyr*
4	*Candida albicans* *Candida krusei*
5	*Candida albicans* *Candida kefyr*
6	*Candida albicans* *Candida glabrata*
7	*Candida tropicalis* *Candida dubliniensis*
8	*Candida glabrata* *Candida tropicalis*

Los resultados obtenidos de las pruebas de sensibilidad frente a los antimicóticos probados (fluconazol, itraconazol y ketoconazol) se muestran en el cuadro 3.

**Cuadro 3:** Concentraciones inhibitorias mínimas probadas para evaluar los patrones de sensibilidad de las especies de *Candida* aisladas de ocho pacientes con candidiasis oral mixta

Aislamiento de candidiasis oral mixta	Especies identificadas	Ketoconazol (µg/ml)	Itraconazol (µg/ml)	Fluconazol (µg/ml)
1	*Candida albicans*	<0,1 S	0,6 R	<4 S
	*Candida krusei*	0,25 SDD	0,8 R	12 SDD
	*Candida glabrata*	1,0 R	>1 R	9 SDD
2	*Candida albicans*	<0,1 S	0,4 SDD	<4 S
	*Candida tropicalis*	<0,1 S	>1 R	<4 S
3	*Candida albicans*	<0,1 S	>1 R	<4 S
	*Candida kefyr*	<0,1 S	<0,1 S	<4 S
4	*Candida albicans*	<0,1 S	0,1 S	<4 S
	*Candida krusei*	0,25 SDD	>1 R	16 SDD
5	*Candida albicans*	<0,1 S	0,6 R	<4 S
	*Candida kefyr*	<0,1 S	0,2 SDD	<4 S
6	*Candida albicans*	0,25 SDD	>1 R	<4 S
	*Candida glabrata*	0,7 R	>1 R	40 R
7	*Candida dubliniensis*	<0,1 S	0,6 R	<4 S
	*Candida tropicalis*	<0,1 S	>1 R	<4 S
8	*Candida tropicalis*	<0,1 S	>1 R	<4 S
	*Candida glabrata*	<0,1 S	0,4 SDD	<4 S

S: sensible; R: resistente; SDD: sensible dosis-dependiente

## Discusión

El diagnóstico de candidiasis oral en pacientes con diabetes mellitus es más frecuente que en la población sana. Existen diversos reportes en los cuales se indica que las cepas de *Candida,* aisladas de pacientes con HIV, diabetes mellitus de tipo 2, cáncer y candidiasis oral, presentan actividad proteolítica que facilita la invasión de los tejidos y el establecimiento de infección localizada o superficial que, en ocasiones, evoluciona a sistémica en comparación con las que presentan los sujetos sin dichas condiciones ^19^.

En este estudio, la candidiasis oral se encontró en 87,5 % (63/72) de los pacientes con diabetes mellitus de tipo 2 estudiados, con predominio de las mujeres sobre los hombres (1,5:1). También, se observó que la candidiasis oral es más frecuente (58,7 %; 37/63) en el grupo de adultos de los 36 a los 59 años; esto puede atribuirse a varios procesos que acompañan el avance de la edad, como la pérdida de dientes ^20^, el uso de prótesis que provocan la reducción del pH salival, favoreciendo la adhesión y el desarrollo de *Candida* spp. ^21^, y como la xerostomía, que implica disminución de la concentración de la saliva y sus elementos antifúngicos (lisozimas, lactoferrinas y lactoperoxidasas)*.* Estos resultados concuerdan con lo publicado por Suárez *et al*. ^12^.

Se encontraron diferencias según el tipo de paciente: hospitalizados y no hospitalizados. En los primeros, fue más frecuente encontrar candidiasis oral ya que, si bien el motivo de la hospitalización fue descompensación metabólica, se agrega la existencia de factores predisponentes, implícitos en su hospitalización prolongada, tales como mala higiene, mal estado nutricional y terapia con antibióticos, los cuales favorecen el desarrollo de la infección, aunque no haya una asociación estadística entre dichas variables. Osuna *et al*. identificaron que el 21% de casos de candidiasis oral se presentó en pacientes cuya causa de admisión al hospital fue descompensación metabólica y que tuvieron una mayor estancia hospitalaria (más de 10 días) ^22^. Fanello *et al*. demostraron que existe un gran riesgo de infección en la cavidad oral conforme aumentan los días de hospitalización y observaron manifestación de la candidiasis oral en los primeros siete días. Ambas publicaciones concuerdan con el motivo de internamiento y el tiempo de evolución de la candidiasis oral que se encontró en los pacientes del presente estudio ^23^.

Existen ciertos desacuerdos respecto a si los factores metabólicos, que constituyen la mejor manera de controlar la enfermedad y evitar el desarrollo de complicaciones macrovasculares y microvasculares, tienen correlación con la predisposición a la candidiasis oral en la población con diabetes mellitus de tipo 2. Carda *et al.* demostraron que pacientes con HbA1C por encima del 8 %, tenían aumento de la glucosa salival, lo que favorece el desarrollo de este tipo de levaduras ^20^. De igual manera, Balan *et al*. afirman que, durante los episodios de hiperglucemia, la alteración ambiental de la cavidad oral aumentó la producción de glucosa y ácido en la saliva, lo que promovió la transición de *Candida* spp. de comensal a agente patógeno ^24^. En el presente trabajo, se encontró una relación positiva entre valores elevados de HbA1C (por arriba de 8,4 %) y candidiasis oral mixta, sin alcanzar una significancia estadística.

Entre los agentes etiológicos identificados, *C. albicans* fue el más aislado (72 %), tal y como se reporta en la mayoría de los artículos relacionados con candidiasis. En un estudio de Mohammadi *et al*., se concluyó que las especies de *Candida* encontradas con mayor frecuencia en la cavidad oral de los pacientes con diabetes mellitus, son: *C. albicans* (36,2 %), *C. krusei* (10,4 %), *C. glabrata* (5,1 %) y *C. tropicalis* (3,4 %), cifras muy similares a las del presente estudio ^13^. Otros autores han mencionado la identificación de *C. lusitaniae*, *C. kefyr*, *C. guilliermondii* y *C. pulcherrima* en la mucosa oral de pacientes total y parcialmente edéntulos (62,3 %) y en la mucosa oral de pacientes diabéticos (37,7 %) ^24^^,^^25^.

Por otra parte, el porcentaje de cepas no-*albicans,* identificado en el presente estudio, fue del 27 %: 11% de *C. tropicalis*, 6 % de *C. glabrata*, 4 % de *C. dubliniensis*, 3% de *C. krusei* y *C. kefyr*. Rosa-García *et al.* reportaron porcentajes semejantes para las cepas de *C. albicans* (74,6 %), *C. tropicalis* (15,2 %) y *C. kefyr* (3,4 %) en un estudio sobre la colonización e infección bucal por *Candida* spp. en pacientes con diabetes mellitus de tipo 2 o sin ella que, además, tenían enfermedad renal crónica con necesidad de diálisis ^26^.

El segundo agente etiológico más aislado (11 %) en este proyecto fue *C. tropicalis* (11 %)*,* que también fue encontrado en segundo lugar por Zuza-Alves *et al*., con una frecuencia del 4,5 %, en un estudio realizado en Brasil, a partir de aislamientos de especies de *Candida* de muestras de la cavidad oral de pacientes con trasplante de hígado ^27^. Es importante mencionar que *C. tropicalis* está cobrando mayor relevancia debido a su frecuencia y a sus factores de virulencia, ya que impactan directamente en la estrategia terapéutica. En un estudio indio, se concluyó que existe un aumento significativo de las infecciones por *Candida* spp. en pacientes con cáncer oral, sometidos a quimioterapia o radioterapia, en quienes predominaron las especies no-*albicans*, principalmente, *C. tropicalis* (42,8 % de los casos) ^27^.

En una revisión sistemática de Reyes-Montes *et al*. sobre el estado actual de la etiología de la candidiasis en nuestro país, se menciona que la forma clínica superficial más común es la genital (47,81 %), seguida por la candidiasis oral (36,26 %) ^28^. Esta forma clínica se presentó en diferentes poblaciones, como pacientes con HIV o sin él, cáncer, diabetes mellitus, niños y adolescentes, niños con mala nutrición y personas de la comunidad Tarahumara.

El agente etiológico más aislado fue *C. albicans* (77,24 %), seguido del complejo *C. glabrata* (13,16 %), *C. tropicalis* (5,13 %), *C. krusei* (3,29 %) y el complejo *C. parapsilosis* (menos del 1%). En este estudio, la frecuencia de aislamientos es muy similar para la especie *C. krusei,* pero no se identificaron especies del complejo *C. parapsilosis* en ninguno de los casos de candidiasis oral mixta.

En el presente estudio, ocho pacientes (13 %) presentaron aislamientos mixtos: un paciente tenía coinfección por tres especies de Candida (*C. albicans*, *C. krusei* y *C. glabrata*) y siete pacientes tenían coinfección por dos especies: *C. albicans - C. kefyr* (dos pacientes), *C. tropicalis - C. glabrata*, *C. dubliniensis - C. tropicalis, C. albicans - C. tropicalis*, *C. albicans - C. glabrata* y *C. albicans - C. krusei*. Estos dos últimos aislamientos también fueron reportados en el 8 % de los pacientes con diabetes mellitus en el estudio de Mohammadi ^13^.

La coexistencia de diferentes especies hace más persistente la infección y puede señalar un estado de riesgo para el desarrollo de infecciones más graves, además de dificultar su tratamiento. De ahí la importancia de la identificación de *C. tropicalis* y *C. glabrata,* especies que en los últimos años han aumentado su patogenicidad, incluso, se ha determinado sinergismo entre ambas especies para causar candidiasis orofaríngea grave ^26^^,^^29^. Estas infecciones son de difícil tratamiento porque *C. tropicalis* y *C. glabrata* son menos sensibles a los azoles, lo cual aumenta el riesgo de candidemia mediante diferentes mecanismos ^24^^,^^26^^,^^29^.

Otros autores han publicado frecuencias de aislamientos mixtos similares a los de este proyecto y con especies parecidas ^12^^,^^13^^,^^30^. De la Rosa-García *et al*. identificaron nueve cultivos mixtos en pacientes con diabetes mellitus de tipo 2, uno de ellos causado por tres especies (*C. albicans*, *C. glabrata* y *C. tropicalis*), cuatro por *C.albicans - C. glabrata* y uno por *C. glabrata - C. tropicalis*^26^*.* Martínez *et al.* señalaron una relación entre el número de especies aisladas de la cavidad oral de pacientes con HIV positivo y el tipo de infección. Afirman que el aislamiento de una sola especie se asocia con pacientes con una infección primaria, mientras que el aislamiento de más de una especie ocurre en aquellos que sufren infecciones recurrentes ^30^. Asimismo, estudios sobre los patrones de recurrencia confirman que la infección producida por una cepa diferente a la que originó el episodio inicial prevalece en pacientes con tratamiento previo de antifúngicos ^30^.

El tratamiento de la candidiasis oral usualmente es de base tópica (nistatina, clotrimazol, ketoconazol) para pacientes que no sufren ningún tipo de complicaciones. Sin embargo, la terapia antimicótica con triazoles es apropiada en pacientes con infecciones recurrentes resistentes al tratamiento o en pacientes con gran riesgo de desarrollar infecciones sistémicas ^31^, como aquellos con HIV, cáncer, diabetes mellitus, etc. Por tal motivo, en el presente estudio se evaluó la sensibilidad *in vitro* de las especies identificadas mediante difusión en agar con fluconazol, itraconazol y ketoconazol.

Cabe mencionar que, en el caso específico del presente trabajo, la respuesta clínica al tratamiento antimicótico dependió de ciertos factores, como las características de inmunidad de los pacientes con diabetes mellitus de tipo 2 (disminución de la población de células dendríticas, eosinófilos y neutrófilos, entre otros) ^32^, incremento de la glucosa salival, los factores de virulencia expresados por *Candida* spp., al igual que la sensibilidad del microorganismo al medicamento y la farmacodinamia de este, entre otros.

En los últimos años, en numerosos estudios se ha reportado que el incremento en la frecuencia de los aislamientos de especies no-*albicans*, la mayoría proveniente de pacientes con algún tipo de inmunosupresión, presenta menor sensibilidad que *C.albicans* e, incluso, resistencia a dos o tres agentes antifúngicos ^12^^,^^13^^,^^29^^,^^33^. En este proyecto, se encontró que dos aislamientos de *C. glabrata* (66,7 %) fueron resistentes a ketoconazol y dos cepas de *C. krusei* (100 %) fueron sensibles según la dosis. Esto difiere de los resultados de *C. albicans*, en los cuales todos los aislamientos resultaron sensibles (cuadro 3). Cabe destacar que la mayoría de las cepas no-*albicans* (tres aislamientos de *C. tropicalis* (100 %), dos de *C. glabrata* (66,7 %), uno de *C. dubliniensis* (100 %) y dos de *C. krusei* (100 %) (cuadro 3), fueron resistentes a itraconazol, al igual que el 66,7 % de los aislamientos de *C. albicans.* Lo anterior no concuerda con lo publicado en la literatura científica. Por ejemplo, Sanitá encontró resistencia en el 93,3 % de los aislamientos de *C. glabrata,* en el 5 % de *los de C. tropicalis* y en el 4 % de los de *C. albicans* (29). Por el contrario, otras especies del no-*albicans* presentan un comportamiento similar al de *C. albicans* y son más sensibles a ciertos azoles.

En el presente estudio, esas especies fueron *C. tropicalis*, *C. dubliniensis* y *C. kefyr,* que tuvieron el 100 % de sensibilidad a fluconazol y a ketoconazol, con concentraciones mínimas inhibitorias bajas, incluso por debajo del extremo inferior del intervalo (cuadro 3).

Idealmente, no solo debería evaluarse la resistencia, sino también el mecanismo involucrado, ya que, en el caso de los azoles (los antimicóticos más utilizados), se ha demostrado resistencia cruzada. Derivado de esto, algunos autores describen que la resistencia a fluconazol tiene como consecuencia la resistencia al itraconazol ^34^. Dicha aseveración no coincide con lo encontrado en el presente estudio, ya que la mayoría de las especies aisladas (a excepción de *C. krusei* y *C. glabrata* con espectro sensible dependiente de la dosis) fueron sensibles a fluconazol. Otros autores también han determinado espectros sensibles a dicho fármaco en especies provenientes de pacientes con candidiasis oral ^29^^,^^33^. Se ha observado que las diferencias en la sensibilidad *in vitro* a fluconazol pueden ser desde el 70 % al 100 % en los aislamientos orales, dependiendo del grupo de pacientes estudiado ^29^.

Como se mencionó, el agente antimicótico ante el cual se observaron más casos de resistencia fue el itraconazol (5/6 especies aisladas). Sin embargo, las pruebas estadísticas no mostraron significancia, así que no hay asociación entre el antimicótico y alguna especie en particular de *Candida*. Manzano-Gayosso *et al*. también reportaron un gran porcentaje (19,9 %) de resistencia al itraconazol de especies de *Candida* causantes de onicomicosis, al evaluar su sensibilidad *in vitro* a diferentes fármacos ^35^.

Este estudio determinó que solo existe asociación significativa entre *C. glabrata* y la resistencia al ketoconazol (66,7 %). Esto concuerda con el gran porcentaje de aislamientos orales resistentes a ketoconazol (47,2 %) que publicó Bremenkamp en un estudio sobre pacientes que padecían diabetes mellitus ^15^. Estos resultados se deben a que el ketoconazol es uno de los tratamientos más indicados para las candidiasis superficiales, lo que se refleja en el gran porcentaje de resistencia; por otro lado, *C. glabrata* es la especie más representativa en el grupo de pacientes adultos. Lindberg *et al*. demostraron con significancia estadística que el aislamiento de *C. glabrata* es más común en pacientes con una edad aproximada de 50 a 60 años, que fue el grupo etario en el que predominó la candidiasis oral ^36^.

Entre los diversos estudios de sensibilidad *in vitro* a antifúngicos en que se incluyen los métodos de microdilución en caldo, citometría de flujo y difusión en disco, es este último el que se considera como el método de referencia por el *Clinical and Laboratory Standards Institute*. En la literatura dermatológica coreana, Song demostró concordancia con dicho método al utilizar las tiras de difusión en agar (E-test®) de tres antimicóticos con especies de *Candida* aisladas de pacientes con candidiasis oral ^37^. Tales resultados fueron similares a los encontrados en el presente estudio.

Si bien no se puede establecer una correlación entre la sensibilidad *in vitro* y la respuesta clínica ^34^, determinar las concentraciones inhibitorias mínimas contribuye a la identificación de cepas sensibles y, aun más importante, a la identificación de cepas resistentes a ciertos agentes antifúngicos, lo cual podría explicar los fracasos observados con diversos tratamientos.

En conclusión, con los hallazgos analizados se estableció que no se encontraron asociaciones estadísticamente significativas que expliquen el desarrollo de candidiasis oral según con la edad, el sexo, el nivel de hiperglucemia, el tiempo de diagnóstico de la diabetes o el tratamiento antihiperglucemiante empleado; o que expongan por qué el 13 % de pacientes descompensados con diabetes mellitus de tipo 2 desarrolló candidiasis mixta en la cavidad oral.

La especie aislada de pacientes descompensados con diabetes mellitus de tipo 2 y candidiasis oral mixta que resultó ser más sensible a los tres antimicóticos evaluados (fluconazol, itraconazol y ketoconazol), fue *C. kefyr* (83,3 %); *C. krusei* (66,7 %) fue sensible según la dosis y la especie con mayor resistencia fue *C. glabrata* (55,6 %), con una relación estadísticamente significativa (p=0,015). El ketoconazol fue el único antimicótico al que se asoció resistencia de una especie (66,7 % de *C. glabrata* versus 0 % de las demás especies) con un valor de p=0,018. Este resultado pone en evidencia la importancia de una adecuada y completa identificación de las especies de *Candida* involucradas en el desarrollo de la candidiasis oral mixta en pacientes con diabetes mellitus de tipo 2.
